# Rational discovery of dual-indication multi-target PDE/Kinase inhibitor for precision anti-cancer therapy using structural systems pharmacology

**DOI:** 10.1371/journal.pcbi.1006619

**Published:** 2019-06-17

**Authors:** Hansaim Lim, Di He, Yue Qiu, Patrycja Krawczuk, Xiaoru Sun, Lei Xie

**Affiliations:** 1 Ph.D. Program in Biochemistry, The Graduate Center, The City University of New York, New York, New York, United States of America; 2 Ph.D. Program in Computer Science, The Graduate Center, The City University of New York, New York, New York, United States of America; 3 Ph.D. Program in Biology, The Graduate Center, The City University of New York, New York, New York, United States of America; 4 Department of Computer Science, Hunter College, The City University of New York, New York, New York, United States of America; 5 Department of Biostatistics, School of Public Heath, Shandong University, Jinan, Shandong, People’s Republic of China; Heidelberg Institute for Theoretical Studies (HITS gGmbH), GERMANY

## Abstract

Many complex diseases such as cancer are associated with multiple pathological manifestations. Moreover, the therapeutics for their treatments often lead to serious side effects. Thus, it is needed to develop multi-indication therapeutics that can simultaneously target multiple clinical indications of interest and mitigate the side effects. However, conventional one-drug-one-gene drug discovery paradigm and emerging polypharmacology approach rarely tackle the challenge of multi-indication drug design. For the first time, we propose a one-drug-multi-target-multi-indication strategy. We develop a novel structural systems pharmacology platform 3D-REMAP that uses ligand binding site comparison and protein-ligand docking to augment sparse chemical genomics data for the machine learning model of genome-scale chemical-protein interaction prediction. Experimentally validated predictions systematically show that 3D-REMAP outperforms state-of-the-art ligand-based, receptor-based, and machine learning methods alone. As a proof-of-concept, we utilize the concept of drug repurposing that is enabled by 3D-REMAP to design dual-indication anti-cancer therapy. The repurposed drug can demonstrate anti-cancer activity for cancers that do not have effective treatment as well as reduce the risk of heart failure that is associated with all types of existing anti-cancer therapies. We predict that levosimendan, a PDE inhibitor for heart failure, inhibits serine/threonine-protein kinase RIOK1 and other kinases. Subsequent experiments and systems biology analyses confirm this prediction, and suggest that levosimendan is active against multiple cancers, notably lymphoma, through the direct inhibition of RIOK1 and RNA processing pathway. We further develop machine learning models to predict cancer cell-line’s and a patient’s response to levosimendan. Our findings suggest that levosimendan can be a promising novel lead compound for the development of safe, effective, and precision multi-indication anti-cancer therapy. This study demonstrates the potential of structural systems pharmacology in designing polypharmacology for precision medicine. It may facilitate transforming the conventional one-drug-one-gene-one-disease drug discovery process and single-indication polypharmacology approach into a new one-drug-multi-target-multi-indication paradigm for complex diseases.

## Author summary

Polypharmacology has emerged as a new strategy for discovering novel therapeutics. Existing efforts in the rational design of polypharmacology have three limitations: focus on a single clinical indication, difficulties in target selection and lead identification/optimization, and ignorance of genome-wide drug-target interactions. Multi-indication therapeutics are needed for complex diseases such as cancer, which have multiple pathological manifestations. The design of multi-indication drugs requires the knowledge of chemical-protein interactions on a genome scale. To enhance our capability of identifying genome-wide chemical-protein interactions, we develop a new structural systems pharmacology platform 3D-REMAP that overcomes the limitations of existing drug-target prediction methods. We propose a strategy that uses the concept of drug repurposing to address challenges in designing dual-indication drugs that can synergistically achieve two desired clinical end points. As a proof-of-concept, we computationally predict and experimentally validate that levosimendan, a PDE inhibitor for heart failure that is associated with all existing anti-cancer therapies, is a kinase inhibitor and active against lymphoma. We further identify biomarkers that predict a patient’s response to levosimendan. This study demonstrates the potential of structural systems pharmacology in designing polypharmacology for precision medicine. Our approach may facilitate transforming the conventional polypharmacology approach to a new one-drug-multi-target-multi-disease paradigm.

## Introduction

Multi-factorial, multi-genic complex diseases such as cancer and Alzheimer’s disease are associated with multiple pathological manifestations. For example, hypertension, inflammation, and herpes virus infection could all be related to the tau and amyloid beta pathologies of Alzheimer’s disease [1–3]. The successful treatments of complex diseases require targeting multiple disease-causing genes that are in either the same or different pathways to achieve additive or synergistic effect, as well as checking drug resistance. In addition, therapeutics may trigger a systematic response that is mediated by on-target or off-target effects, leading to serious side effects. For example, almost all of chemotherapy, targeted therapy, and immunotherapy for cancer treatment increase the risk of heart failure [4, 5]. Thus, an ideal therapy should be not only effective on multiple clinical indications but also able to mitigate side effects.

Recently, multi-targeted therapy (also known as polypharmacology) through either drug combination or a single polypharmacological agent has emerged as a new paradigm of drug discovery. It is argued that single-agent polypharmacology has advantages over the drug combinations [6]. In spite of serendipitous success of polypharmacology, two major challenges remain in the rational design of polypharmacology: target selection and lead compound identification. With regards to the target selection, existing polypharmacology drug design mainly focuses on multiple targets that are involved in a single disease, i.e. a one-drug-multi-target-one-disease paradigm. However, it is challenging to select the right target combinations for the disease of interest due to our limited understanding of gene-disease associations. In terms of lead identification, existing medicinal chemistry efforts typically combine two distinct molecular scaffolds that have desired activity towards each target of interest into a single chemical entity through merging, fusing, or linking two molecules [7–9]. This approach often leads to molecules with poor drug-likeness properties [10]. As a result, the subsequent lead optimization is often a serious bottleneck in the polypharmacology. Furthermore, two additional challenges have not been addressed by existing polypharmacology paradigm. It rarely takes genome-wide drug-target interactions and multi-indications into consideration.

Here, we develop a different strategy for the design of polypharmacology. Our method is novel in both concept and methodology. Conceptually, we aim to target two different clinical indications of interest at the same time, specifically, heart failure and cancer in this study. Thus, our strategy could transfer the conventional one-drug-multi-target-one-disease approach to a new one-drug-multi-target-multi-indication paradigm that is needed but has not been systematically explored for the treatment of complex diseases. Methodologically, we utilize the concept of drug repurposing instead of screening compounds against multiple targets then fusing them to address challenges in the target selection and the lead identification. We start with approved drugs that are used for the treatment of one indication of interest. Then, we search for their potential use for the second indication of interest. On one hand, it is possible for us to identify genome-wide target profiles of multi-target drugs. On the other hand, the time and cost of multi-target drug development from lead optimizations to clinical trials could be significantly reduced because the pharmaceutical profiles (e.g. ADME, toxicology etc.) of the compounds are already optimized for one of the indications. It is noted that this approach is different from the conventional drug repurposing that rarely takes into account of the synergy between primary indication and repurposed indication.

Our method is based on the premise that a drug often interacts with unintended targets, i.e., off-targets. These off-targets may cause unwanted side effects (i.e., anti-target) or function as therapeutic targets for other diseases. The objective of our method is to identify such targets and associated marketed drugs that can selectively interact with multiple targets. The primary target(s) and their off-targets could collectively reduce side effect and enhance therapeutic effects. Selective promiscuity has been actively pursued in kinase inhibitor design [11]. However, most of the current efforts in multi-target screening only consider the binding promiscuity within a single gene family. The multi-indication drug design strategy needs to extend the concept of selective promiscuity across gene families. It is challenging to identify the genome-wide drug-target interactions [12]. In this study, we develop a novel platform, 3D-REMAP, for screening off-targets of marketed drugs on a genome scale. 3D-REMAP integrates diverse chemical genomics, structural genomics, and functional genomics data as well as combines various computational tools from bioinformatics, chemoinformatics, biophysics, and machine learning. Although chemical genomics data provide a rich resource for the development of machine learning model of genome-scale chemical-protein interaction prediction, they are still noisy, incomplete, and biased. The key idea of 3D-REMAP is to apply 3-dimensional ligand binding site comparison and protein-ligand docking to augment chemical-protein interaction data. Then machine learning model is trained using the structure-augmented chemical genomics data. As a result, 3D-REMAP partially overcomes the limitations of each individual data set and method. As evidenced by the experimentally validated predictions in this paper, 3D-REMAP outperforms state-of-the-art bioinformatics, chemoinformatics, protein-ligand docking, and machine learning methods alone.

As a proof-of-concept, we apply the proposed methodology to design dual-indication anti-cancer therapy. The repurposed drug can both demonstrate anti-cancer activity for cancers that do not have effective treatment and reduce the risk of heart failure that is associated with all types of existing anti-cancer therapies. In spite of the tremendous success of kinase-targeting drugs and immunotherapy in the treatment of cancers, the development of anti-cancer targeted therapeutics faces three challenges: serious side effects, especially, cardiotoxicity, variable drug responses, and acquired drug resistance. Regarding the cardiotoxicity of anti-cancer therapy, it has been associated with all types of chemotherapy, targeted therapy, and immunotherapy. For example, tyrosine kinase-targeting drugs are shown to be associated with a higher risk of onset heart failure in adult cancer patients [4]. Recently, fatal heart failures have been reported in patients treated with immune checkpoint inhibitors [5]. With regards to therapeutic efficacy, the clinical responses of immune checkpoint inhibitors strongly depend on the interplay of microenvironment and global immunity [13]. As a result, only a small portion of cancer patients respond to immunotherapy. Similarly, the efficacy of kinase-targeting cancer therapeutics depends on the catalytic activity of the targeted kinase [14]. Existing kinase drugs mainly involve targeting a kinase that is directly activated by mutation. Due to the high heterogeneity of cancers, many patients may not harbor the activated mutation of targeted kinases. Thus, it is still urgently needed to target other cancer mechanisms as well as identify patients associated with such aberrations for effective and precision anti-cancer therapy. In addition to primary resistance to immunotherapy and kinase-targeted drugs, adaptive and acquired resistance to anti-cancer therapies inevitably emerges [15, 16], even in cancer patients who initially respond to the therapeutics. Acquired resistance is mediated by multiple mechanisms such as modification of targeted kinases, activation of bypass signaling pathways, or histological transformation. Drug combinations that can target multiple cancer pathways has been actively pursued to combat the drug resistance in the treatment of cancer [17]. Although the multi-targeted therapy has been discovered to either mitigate side effect [18] or enhance therapeutic efficacy alone [19], few studies have been reported to design molecules as a dual-indication agent that can achieve both of objectives at the same time. In this study, we for the first time discover the multi-target dual-indication agent for the precision anti-cancer therapy.

Using 3D-REMAP, we have made several innovative discoveries. First, we discover that levosimendan, a drug for heart failure, is a novel inhibitor of serine/threonine-protein kinase RIOK1 and a number of other kinases. Second, we uncover that RIOK1 and its associated RNA processing pathway is an effective novel target for multiple types of cancers, especially, lymphoma. Different from existing targeted kinases that harbor the activated mutation, the aberration of RIOK1 is mainly associated with its overexpression. Thus, targeting RIOK1 may provide new opportunities in the cancer treatment. Third, we suggest that levosimendan can be a novel lead compound for developing a more safe and effective anti-cancer therapy to overcome the cardiotoxicity and the drug resistance of existing kinase-targeting drugs, either as a single polypharmacological agent or a component in a drug combination. Our findings may have significant implications in anti-cancer drug discovery and demonstrate the potential of the genome-wide multi-target screening in precision drug discovery.

## Results

### Rational polypharmacology strategy for discovering dual-indication cross-gene family multi-target agents

**Fig 1:**
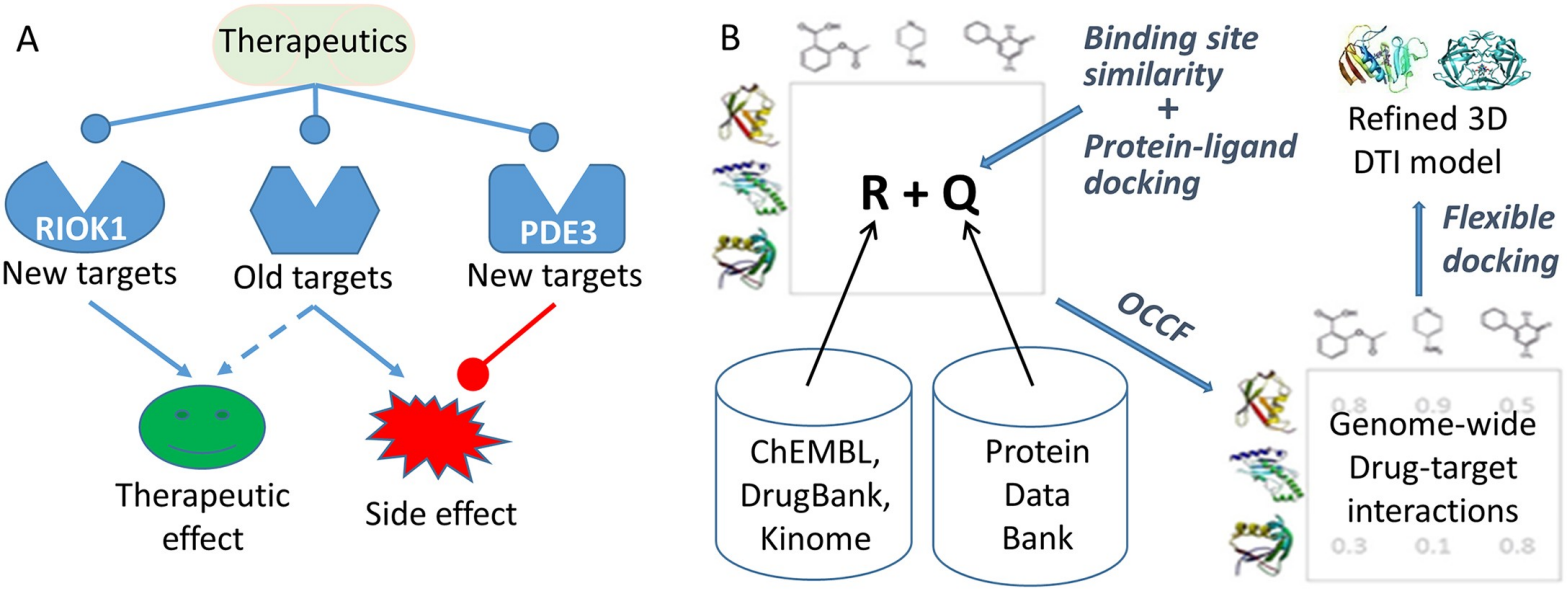
3D-REMAP concept figure. (A) A one-drug-multi-target-multi-indication strategy to screen drugs that can both enhance therapeutic effect and mitigate side effect. (B) Schema of 3D-REMAP, a multi-target screening platform that integrates structural genomics and chemical genomics data and combines tools from bioinformatics, chemoinformatics, protein-ligand docking, and machine learning. *R* and *Q* denote observed protein-chemical interactions in chemical genomics databases, and predicted protein-chemical interactions from ligand binding site similarity coupled with protein-ligand docking, respectively. These two matrices are the input for the machine learning algorithm weighted imputed neighborhood-regularized One-Class Collaborative Filtering (winOCCF) to predict genome-wide drug-target interactions. See Method section for details. DTI: drug-target interaction.

The rationale of our multi-target drug screening strategy is that the serious side effect caused by therapeutic target(s) (i.e. on-target effect) or anti-target(s) (i.e. off-target effect) of a drug can be mitigated by its or another drug’s interaction with an off-target, which is against the side effect, as shown in Fig 1A. The drug-off-target interaction can come from a single polypharmacological agent or multiple components in a drug combination. In this study, we focus on searching for marketed drugs that may act as a dual-indication agent that can mitigate the cardiotoxicity of anti-cancer therapy, at the same time, present anti-cancer potency. Contemporary anti-cancer therapeutics, such as tyrosine kinase inhibitors, anthracycline chemotherapy, and immunotherapy, are all associated with the cardiotoxicity [20].

### Hypothesis generation using 3D-REMAP, a genome-scale multi-target screening platform

The rational discovery of the dual-indication therapeutic agent was achieved using a novel genome-scale multi-target screening platform, 3D-REMAP, as illustrated in Fig 1B. 3D-REMAP framework applied here integrates diverse data from structural genomics and chemical genomics, as well as synthesized tools from bioinformatics, chemoinformatics, biophysics, and machine learning. Since each data set alone is biased, incomplete, and noisy, and a single computational or experimental tool has its inherited advantages and limitations, the integrative analysis may provide more comprehensive and reliable results. 3D-REMAP takes four networks as input: a protein-chemical interaction (PCI) network, which is represented by the matrix *R*. In this study, the matrix *R* contains 650,581 positively associated chemical-protein pairs for 1,656,274 unique chemicals and 9,685 unique target proteins. In addition to *R*, two other input networks are a chemical-chemical similarity network and a protein-protein similarity network (not shown in Fig 1B). The details in the construction of PCI, chemical-chemical similarity, and protein-protein similarity networks are presented in the Materials and Methods section.

Since observed PCI from chemical genomics is highly sparse, one of the unique features of 3D-REMAP is to apply ligand binding site similarity search and protein-ligand docking to screen off-targets of given drugs across human structural proteome [21–27]. The putative drug-off-target interactions serve as imputations to fill in the unobserved entries in the matrix *R*. The structural genomics data are complementary with the chemical genomics data and may provide additional information on genome-wide PCIs. The optimal ligand binding site similarity search requires to select a primary target that has an experimentally solved structure or a high-quality homology model as well as a defined binding pocket as a template for the search. In this study, five clinically used PDE3 inhibitors (milrinone, anagrelide, amrinone, enoximone, and levosimendan) [28] that are used for the treatment of heart failure were selected as our starting point. Using the PDE3B structure as a template (PDB ID 1SO2), potential off-targets of PDE3 inhibitors were predicted by comparing the binding site of PDE3B with 10,472 non-redundant human protein structures using SMAP software [29–31]. Their interactions with the five drugs were predicted using protein-ligand docking [32]. Table 1 shows the top 20 predicted PDE3B off-targets and their docking scores with five drugs. The putative drug off-target interactions (SMAP p-value less than 2.0e-3 and docking score less than -7.5) forms the matrix *Q* and fills in the part of unobserved entries in the PCI network.

**Table 1:** Top 20 ranked putative off-targets of PDE3B. Docking score that is less than -7.5 is highlighted in bold.

PDB	Uniprot	Protein	SMAP *p*-value	Protein-Drug Docking Score				
				Milrinone	Anagrelide	Levosimendan	Amrinone	Enoximone
5U09	P21554	Cannabinoid receptor	4.13e-4	-7.4	-7.3	**-9**	-7.1	**-7.6**
1XU9	P28845	Corticosteroid 11β dehydrogenase	4.16e-5	**-8.2**	**-7.6**	**-8.8**	-7.1	-7.5
3HX3	P12271	Retinaldehyde-binding protein 1	5.42e-4	-7.3	**-7.6**	**-8.8**	-7	**-7.6**
1R5L	P49638	**α**-tocopherol transfer protein	3.40e-5	-7.2	**-7.8**	**-8.6**	-6.6	-7.1
3VW7	P25116	Proteinase-Activated Receptor 1	6.86e-5	**-7.7**	**-7.8**	**-8.4**	-7.2	-7.5
3SOA	Q9UQM7	Calcium/calmodulin-dependent kinase (CAMK2A)	3.66e-4	-7.6	-6.9	**-8**	-7	-6.9
1UW5	Q00169	Phosphatidylinositol transfer protein	4.36e-4	-7.1	-6.8	**-8**	-6.9	-6.8
3K1Z	Q9BSH5	Haloacid dehalogenase-like hydrolase domain-containing Protein 3	2.63e-4	**-8.2**	**-8.4**	**-8**	**-7.7**	-7.5
4Q6R	O95470	Sphingosine-1-phosphate lyase 1	3.62e-4	-6.4	-7.3	**-8**	-6.4	-6.6
4OQA	P09874	Poly [ADP-ribose] polymerase 1	3.47e-6	-7.1	**-7.8**	**-7.8**	-7	-7
2OBD	P11597	Cholesteryl ester transfer protein	1.43e-3	-7	-7.2	**-7.8**	-6.4	-6.8
2CW6	P35914	Hydroxymethylglutaryl-CoA lyase	1.36e-3	-6.5	-7.3	**-7.8**	-6.3	-7.7
4OQV	Q02127	Dihydroorotate dehydrogenase	3.29e-4	**-8**	-6.7	**-7.8**	-7	-8.2
5KDI	Q96JA3	Pleckstrin homology domain-containing family A protein	9.93e-4	-7.3	-7.2	**-7.6**	-6.6	-7.1
4FC7	Q9NUI1	Peroxisomal 2,4-dienoyl-CoA reductase	1.36e-3	-6.6	-7	**-7.6**	-6.3	-6.5
4OTP	Q9BRS2	Serine/threonine protein kinase (RIOK1)	1.45e-3	-7.0	-7.2	**-7.6**	-6.7	-6.7
5HZ8	P15090	Fatty acid-binding protein	1.08e-3	-7.1	**-7.9**	-7.5	-6.9	-6.7
4P8V	Q15782	Chitinase-3-like protein 2	6.65e-4	-7.3	-6.9	-7.5	-6.6	-6.7
5FYQ	P62826	NAD-dependent protein deacetylase	2.64e-4	-6.2	-6.7	-7.4	-5.9	-6.2
2ONI	Q96PU5	E3 ubiquitin-protein ligase NEDD4-like protein	1.03e-3	-7	-6.6	-7.2	-6.7	-5.7

Using the matrix *R* and *Q* along with chemical-chemical and protein-protein similarity networks as input, a weighted imputed neighborhood-regularized one-class collaborative filtering (winOCCF) algorithm developed by our group [33], was then applied to predict the binding profile of the given drug against the 9,685 targets. To reduce the impact of the potential false positives from the off-target prediction, a confidence weight could be supplied to quantify the uncertainty of the prediction. Thus, 3D-REMAP is robust to noisy data. Finally, atomic details of the prioritized drug-target interactions are analyzed for lead optimization using flexible protein-ligand docking. Because 3D-REMAP integrates chemoinformatics, SMAP, protein-ligand docking, and machine learning and is scalable to tens of thousands of protein targets on a genome scale, it may predict off-targets that are overlooked by protein structure-based approaches and other chemoinformatics methods [33, 34].

### Protein kinases are predicted to be the off-target of PDE3 inhibitors

Among those off-targets predicted by 3D-REMAP, we focused on protein kinases, since a significant number of them are known to be associated with cancers. In addition, it is relatively easy to experimentally validate the predicted off-targets across human kinome. Levosimendan, a marketed drug in Europe and Asia for heart failure, was selected for the initial experimental validations because it shows better protein-ligand docking scores on kinases than the other four drugs (Table 1). The top three predicted kinase off-targets of levosimendan are CAMK2, RIOK1, and FLT3. Additional top-ranked predictions include RIOK3, MYLK4, LTK, CDK7, CDK8, DYR1B, GSK3A, GSK3B, and MAP3K5. The expectation values of all of these predictions are less than 1.0e-11.

### RIOK1 and several other kinases are the off-targets of levosimendan

**Fig 2:**
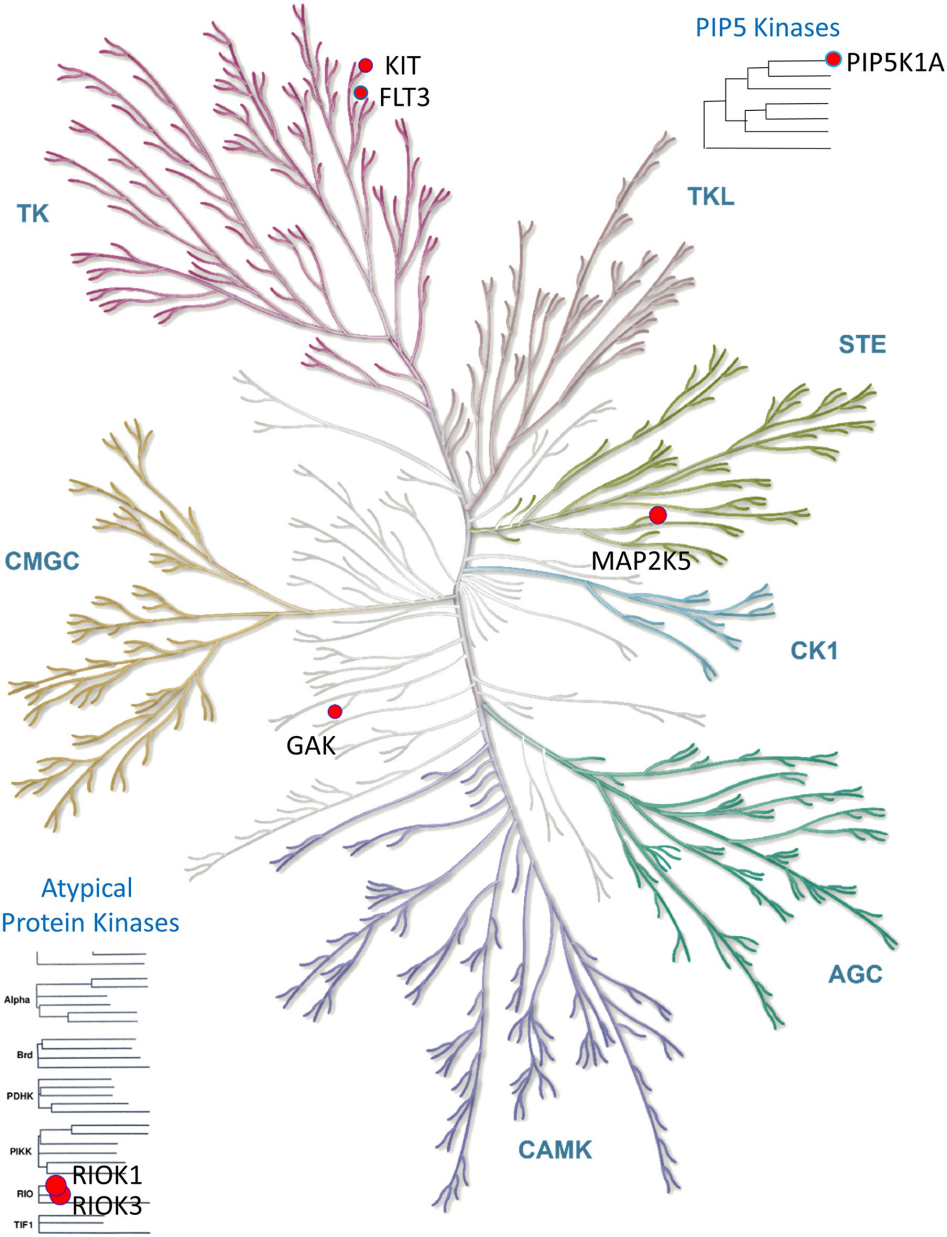
The distribution of kinase off-targets of levosimendan in the human kinome. The off-targets are marked by red circles. The diameter of the circles approximately corresponds to the binding strength. Illustration reproduced courtesy of Cell Signaling Technology, Inc. (www.cellsignal.com/).

KinomeScan assay across 452 human kinases verified our computational prediction (S1 Table). RIOK1 is one of the strongest inhibited kinases by levosimendan. The percentage control of RIOK1 is 15.0 and 0.0 under the treatment of 10 μM and 100 μM of levosimendan, respectively. The dose-response curve further shows that the *K*_d_ of levosimendan against RIOK1 is 0.82 μM. RIOK3, the closest homolog of RIOK1, showed the similar binding strength as RIOK1. In addition, levosimendan also inhibits a number of other kinases besides RIOK1 and RIOK3. The percentage controls of five kinases: FLT3, MAP2K5, PIP5K1A, GAK, and KIT are less than 30.0 under the treatment of both 10 μM and 100 μM of levosimendan. Their distributions in the kinome tree is shown in Fig 2. Both FLT3 and KIT are tyrosine protein kinases. Their inhibitors have recently been approved for the treatment of Acute Myeloid Leukemia and other types of cancers, but a combination therapy is needed to overcome rapidly emerged drug resistance, thus improve patients’ drug responses [35, 36]. Other kinases inhibited by levosimendan belong to serine/threonine protein kinase family (RIOK1, RIOK3, GAK, and MAP2K5) and lipid kinase family (PIP5K1A). In addition to these 7 kinases, levosimendan can moderately inhibit other 29 kinases (percentage of control larger than 30.0 under 10 μM of treatment but less than 30.0 under 100 μM of treatment). As a result, levosimendan may modulate different cancer pathways from those by the tyrosine protein kinase inhibitors, and it could be a novel lead compound for a new anti-cancer therapy to overcome drug resistance of existing drugs or to target different types of cancers through polypharmacology or drug combination.

**Fig 3:**
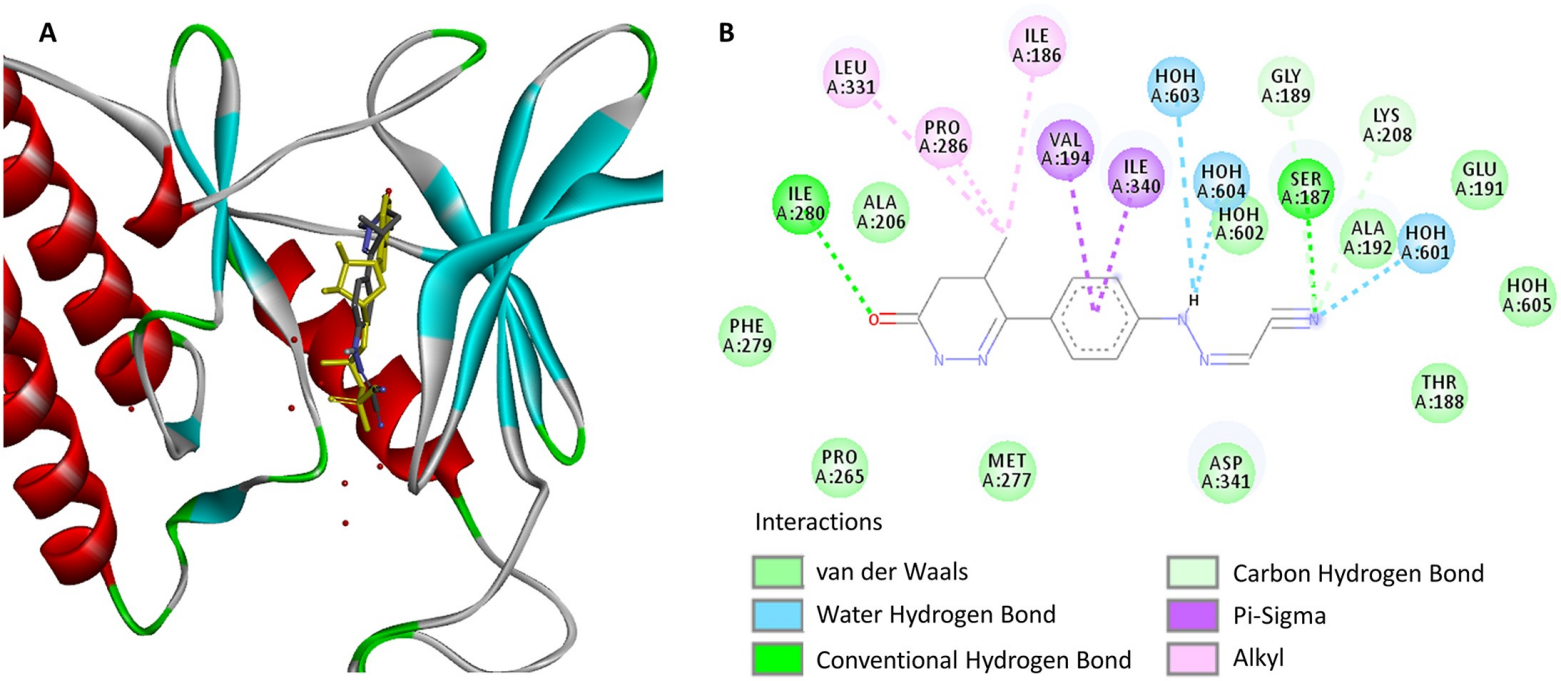
(A) Predicted binding poses of levosimendan (blue stick) and co-crystallized ADP (yellow stick) on RIOK1 (ribbon model). (B) Interaction pattern of levosimendan with RIOK1.

To understand the molecular details in how levosimendan interacts with RIOK1, we re-docked levosimendan into the ATP binding site of RIOK1 using AutodockFR, a flexible receptor protein-ligand docking software [37]. The binding pose and interaction pattern between levosimendan and RIOK1 is shown in Fig 3A and 3B, respectively. The binding pose of levosimendan overlaps that of ADP. Several key interactions between ADP and RIOK1 observed in the crystallized complex structure (PDB ID: 4OTP) are conserved in the levosimendan-RIOK1 complex. They include the hydrogen bonds formed by ILE280, SER187, and water molecules as well as pi-alkyl interaction formed by VAL194. Therefore, levosimendan could inhibit RIOK1 in an ATP-competitive manner. Such information may facilitate the medicinal chemistry efforts in optimizing levosimendan to be a more potent and selective RIOK1 inhibitor. For example, a functional group of hydrogen bond donor may be added to its benzene ring to form hydrogen bond interaction with a crystallized water molecule similar to that in ADP. Amino acid mutations in the binding site may impact the ligand binding. No amino acid mutations in TCGA [38] and COSMIC [39] are observed in the key residues involved in the binding of levosimendan. Thus, levosimendan may target the aberration of RIOK1 that is associated with overexpression or post-translational modification of protein rather than amino acid mutations, distinguishing itself from existing kinase inhibitors.

Comparing the computationally predicted kinase off-targets of levosimendan with the experimentally determined ones across human kinome, we correctly predicted two off-targets out of the top three ranked predictions (RIOK1 and FLT3). Among the top twelve ranked predictions, three predicted off-targets have relatively strong binding affinities to levosimendan (RIOK1, RIOK3, and FLT3), two have intermediate binding affinities (MYLK4 and CAMK2), and others do not have any observed inhibitory activities (LTK, CDK7, CDK8, DYR1B, GSK3A, GSK3B, and MAP3K5). There are four false negatives (MAP2K5, PIP5K1A, GAK, and KIT). There is no doubt that the performance of 3D-REMAP needs to be further improved. Nevertheless, the successfully predicted off-targets of levosimendan from 3D-REMAP cannot be achieved by state-of-the-art protein-ligand docking (PLD), ligand-based virtual screening, and winOCCF (without the matrix *Q* input) alone. As shown in Table 2, 3D-REMAP significantly outperforms all of the conventional methods when evaluated with the predicted and experimentally determined binding profile of 452 kinases. For the top three predictions of nine relatively strong binders, the precision and recall of 3D-REMAP are 66.6% and 22.2%, respectively. The precision and recall for the other methods are all 0.0. Detailed results of protein-ligand docking and ligand similarity search are in S2 and S3 Tables, respectively.

**Table 2:** Comparison of the performance of 3D-REMAP with other methods when predicting that kinase off-targets of levosimendan, which are ranked at the top 2.5%.

	TP	FP	TN	FN	Precision (%)	Recall (%)	FPR (%)
3D-REMAP	4	8	408	32	33.3	11.1	1.92
winOCCF^a^	0	12	404	36	0	0	2.88
PLD	0	12	404	36	0	0	2.88
Binding site similarity + PLD	1	11	405	35	8.33	2.78	2.64
Ligand similarity [40]	0	12	404	36	0	0	2.88

The true positive of the off-target is defined as the kinase with the percentage controls less than 30.0 under the treatment of 100 μM of levosimendan. **PLD**: Protein-Ligand Docking, **FPR**: False Positive Rate, **TP**: True Positive, **FP**: False Positive, **TN**: True Negative, **FN**: False Negative.

^a^The inputs of winOCCF do not include the predicted drug off-target network of PDE3B. A fixed value of 0.1 is used for the matrix *Q* in Fig 1.

### Levosimendan can inhibit the proliferation of multiple types of cancer cells

**Fig 4:**
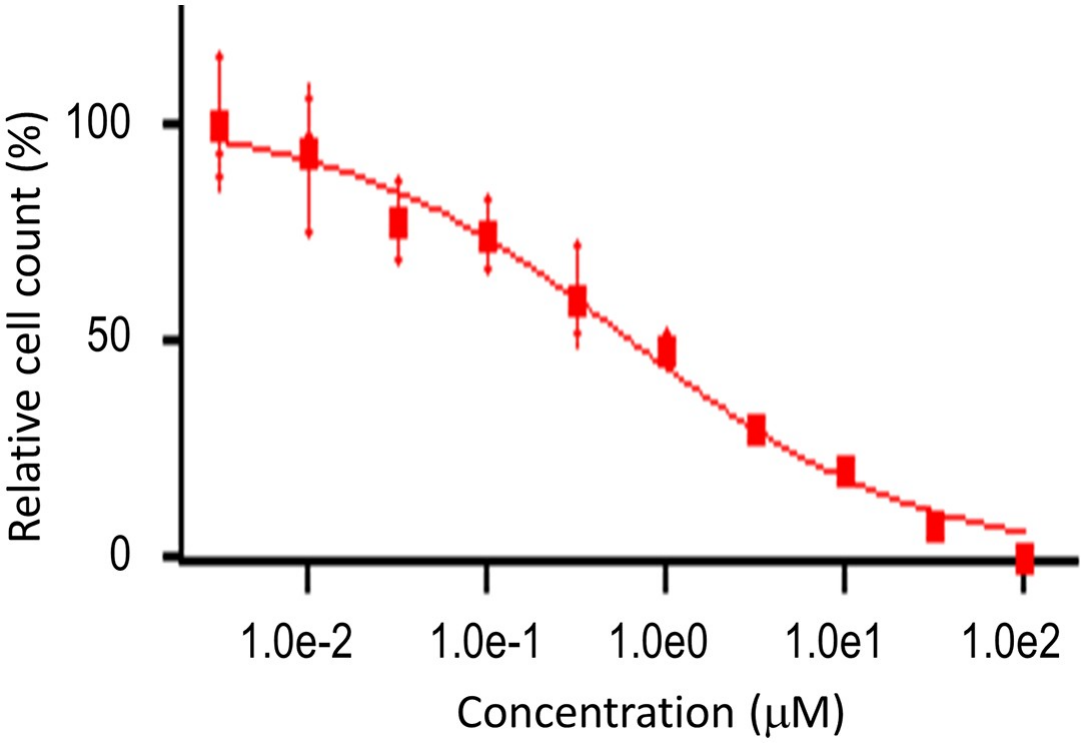
The drug dose response curve of lymphoma SU-DHL-8 cell line under the treatment of levosimendan.

The putative anti-cancer activity of levosimendan was tested for over 200 cancer cell lines across nineteen cancer types (sites of primary tumor), which include bladder, breast, central nervous system, colon, endocrine, eye, female genitourinary system, head and neck, hematopoietic, kidney, liver, lung, pancreas, placenta, prostate, skin, soft tissue, stomach, and testis (S4 Table). Among them, the EC_50_, IC_50_, and GI_50_ of seventeen cell lines were all less than 10.0 μM. Hematopoietic Lymphoma was most sensitive to levosimendan. Four out of the seventeen sensitive cell lines belong to the lymphoma cancer type. Notably, the EC_50_, IC_50_, and GI_50_ for SU-DHL-8 cell line are 0.604 μM, 0.604 μM, and 0.512 μM, respectively. Fig 4 shows the dose response curve of the SU-DHL-8 cell line under the levosimendan treatment. The cell count activity area is 5.0, significantly higher than those of other tested cell lines. Other cancer types sensitive to levosimendan include stomach carcinoma, endocrine carcinoma, kidney tumor, colorectal carcinoma, bladder carcinoma, osteosarcoma, melanoma, prostate hyperplasia, and sarcoma. Thus, levosimendan is a promising lead compound for designing polypharmacological agent or drug combination to treat multiple types of cancers, notably, lymphoma.

### Anti-cancer activity of levosimendan may come from its modulation of RNA processing pathway through the inhibition of RIOK1

Differential gene expression profile analysis across 200 cancer cell lines supports that the anti-cancer activity of levosimendan results from its inhibition of RIOK1. First, RIOK1 is overexpressed in the drug sensitive cell lines. Second, Student’s t-test analysis of gene expression profile identified 475 genes including RIOK1 that contribute to the cell line sensitivity to levosimendan with *q*-value less than 1.0e-3 (S5 Table). Third, gene set overrepresentation analysis suggests that the drug-sensitive genes are significantly enriched with the biological processes of Gene Ontology [41]: rRNA processing, translation, and ribosome biogenesis (FDR<1.0e-3), as shown in Table 3. In addition, the only overrepresented KEGG pathway is “ribosome” (FDR = 6.62e-40). It is established that the primary function of RIOK1 is involved in rRNA processing of ribosome [42, 43]. Thus, differential gene expression and gene set overrepresentation analyses are consistent with our computational prediction and kinase binding assay. It is reasonable to hypothesize that the inhibition of RIOK1 by levosimendan is directly responsible for its anti-cancer activity.

**Table 3:** Overrepresented GO biological process terms responsible for the anti-cancer sensitivity of levosimendan.

GO Term	*p*-value	FE^a^	Bonf^a^	Benj^a^	FDR^a^
GO:0006364. rRNA processing	6.35E-50	16.75	5.68E-47	5.68E-47	9.89E-47
GO:0006413. translational initiation	1.49E-48	22.29	1.33E-45	6.65E-46	2.31E-45
GO:0006614. SRP-dependent co-translational protein targeting to membrane	7.32E-47	28.24	6.55E-44	1.64E-44	1.14E-43
GO:0019083. viral transcription	1.60E-46	24.89	1.43E-43	2.86E-44	2.49E-43
GO:0006412. translation	3.16E-39	12.85	2.83E-36	4.72E-37	4.92E-36
GO:0002181. cytoplasmic translation	6.02E-08	21.24	5.39E-05	7.70E-06	9.38E-05
GO:0042274. ribosomal small subunit biogenesis	7.60E-08	29.04	6.80E-05	8.50E-06	1.18E-04

^a^**FE**: Fold Enrichment, **Bonf**: Bonferroni correction, **Benj**: Benjamini-Hochberg correction, **FDR**: False Discovery Rate

Differential expression analysis also unveiled genes that were significantly associated with resistance to levosimendan. The top genes in this set are involved in a wide variety of cellular functions, including signal transduction, mitosis, cytoskeletal regulation, ion transport, and drug metabolism, but no biological processes or pathways are overrepresented.

Different from gene expression profiles, amino acid mutations and copy number variations associated with the sensitivity and resistance of levosimendan are statistically insignificant. This is consistent with the predicted binding pose of levosimendan in RIOK1. Thus, levosimendan may represent a new class of kinase inhibitors that do not depend on targets activated by mutations.

### Pharmcogenomics modeling for the anti-cancer activity of levosimendan

Using the expression values of genes that are responsible for the sensitivity and resistance of levosimendan as features, we apply a novel feature selection method Kernel Conditional Covariance Minimization [44] to develop machine learning models for the prediction of other cancer cell lines or patients that may respond to levosimendan. We develop two models using CCLE [45] and GDSC [46] data separately. The Spearman’s correlation coefficients for these two models are 0.7547 and 0.6727, respectively. In the leave-one-out cross-validation, the predicted activity area for the most active SU-DHL-8 cell line is around 2.0. Thus, the predicted activity area of 2.0 is used as a threshold for the prediction. S6 Table lists the top fifty ranked predictions.

Top thirty ranked cell lines that are predicted with consensus by both CCLE and GDSC models included JM1 (B cell lymphoma), NU-DUL-1 (B cell lymphoma), SU-DHL-5 (B cell lymphoma), and ALL-SIL (T-Cell Leukemia). Their predicted active areas are larger than 3.8 and 2.0, respectively. It is clear that B-cell lymphoma dominates the sensitive cell lines to levosimendan.

**Fig 5:**
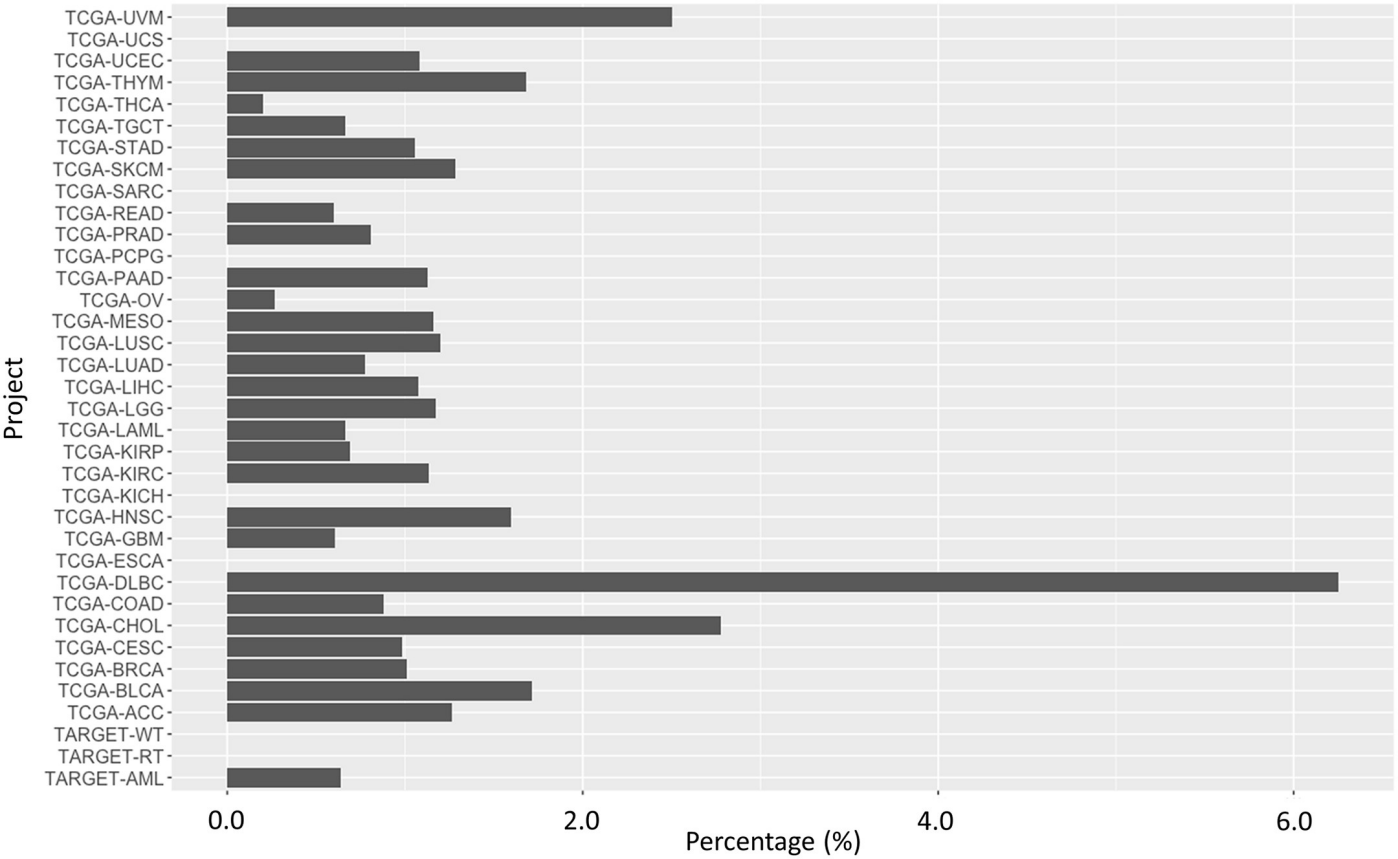
Percentage of cases that are ranked within top 100 in the predictive model over all cases in the TCGA project. The most statistically significant overrepresented cancer type is B-cell lymphoma (TCGA-DLBC).

We further apply the trained CCLE model to predict the cases in TCGA [38] that could respond to levosimendan (S7 Table). Consistent with the cell line assays and predictive models, the top one hundred ranked cases are overrepresented by B-cell lymphoma (TCGA-DLBC) with the *p*-value of 1.05e-2, as shown in Fig 5. The predicted activity areas for the cases of B-cell lymphoma span a broad range from less than 0.2 to larger than 2.0, suggesting that only a portion of B-cell lymphoma patients may respond to the treatment of levosimendan. Except B-cell lymphoma, other TCGA projects are not significantly overrepresented in the top one hundred ranked predictions. However, a number of cases in multiple cancer types have the predicted active area larger than 2.0 and fall into the cancer types that responded to levosimendan treatment in the cell line assay. Notably, they include stomach carcinoma (TCGA-STAD), kidney renal clear cell carcinoma (TCGA-KIRC), prostate adenocarcinoma (TCGA-PRAD), colon adenocarcinoma (TCGA-COAD), bladder urothelial carcinoma (TCGA-BLCA), and skin cutaneous melanoma (TCGA-SKCM). Thus, some patients diagnosed with these cancers may also benefit from the treatment of levosimendan. Due to the heterogeneity of cancers, it is necessary to develop an accurate pharmacogenomics model for the development of levosimendan as a precision anti-cancer therapy.

## Discussion

In this study, we computationally predicted and experimentally validated that levosimendan—a marketed drug for heart failure—can inhibit the growth of multiple cancer cell lines, notably, lymphoma. The anti-cancer activity of levosimendan mainly origins from the modulation of RNA processing pathway by the inhibition of atypical kinase RIOK1. RIOK1 represents a new anti-cancer drug target [47, 48], and the chemical space of its inhibitors has just emerged. Tyrosine kinase inhibitors have been approved in the treatment of lymphoma [49, 50]. Our findings suggest that levosimendan could be used in the combination therapy or as a potential lead compound for new multi-targeted drugs for lymphoma. On one hand, levosimendan may be combined with other tyrosine kinase inhibitors that are associated with the risk of heart failure. Different from inhibitors generated from high-throughput screening or *de novo* design from a single target, it is known that levosimendan interacts with other proteins that are the drug targets for the heart failure than RIOK1. The combination of levosimendan and the tyrosine kinase inhibitor may not only reduce the cardiotoxicity of the tyrosine kinase inhibitor but also enhance the anti-cancer efficacy since they act on different cancer pathways. On the other hand, levosimendan interacts with multiple kinases that are associated with cancers as well as proteins that are responsible for heart failure. The binding promiscuity of levosimendan may allow us to use it as a lead compound to design a new type of dual action agent by modulating multiple targets that are involved in both side effects and disease mechanisms. In many cases, disease-causing genes have pleiotropic effects on biological system, thereby making on-target side effect(s) unavoidable. In contrast with the conventional drug discovery process that designs highly selective ligands, it is possible to mitigate the side effect by designing a drug to bind an off-target that is against the side effect [18].

To further advance the potential of levosimendan in cancer treatment, several points remain to be investigated. First, the anti-cancer potency of levosimendan can be further improved by designing personalized derivatives. The binding pose analysis may provide valuable clues to the drug design. Second, our machine learning model predicted that several other cell lines are sensitive to levosimendan. A patent has shown that PDE3 inhibitors may be active against HeLa cell line [51]. It will be interesting to test the sensitivity of levosimendan and other PDE3 inhibitors on more cancer types. Finally, *in vivo* anti-cancer activity of levosimendan need to be verified.

The rational design of dual-indication multi-targeted drugs is an extremely challenging task. It requires modeling drug actions on a multi-scale, from genome-wide drug-target interactions to system level drug responses. This study showcases that 3D-REMAP is a potentially powerful tool towards designing polypharmacology and drug repurposing. 3D-REMAP provides a framework to integrate heterogeneous data from chemical genomics, structural genomics, and functional genomics, and synthesize diverse tools from bioinformatics, machine learning, biophysics, and systems biology for the multi-scale modeling of drug actions. An emerging paradigm of systems pharmacology enables the understanding of cellular mechanism of drug action at the organismal level, but it lacks the power to screen and design new chemical entities. Structure-based drug design has been successful in discovering novel drug molecules with fine-tuned binding properties to specific targets. However, the designed drug-target interaction may not transform well into desired organismal level drug response. 3D-REMAP may bridge the structure-based drug design and systems pharmacology; thus, it facilitates drug discovery for complex diseases. In spite of the success in this proof-of-concept study, many aspects of 3D-REMAP could be improved. First, the prediction accuracy of each individual algorithm, such as protein-ligand docking and protein-chemical interaction prediction in the computational pipeline needs to be improved. Second, we use winOCCF to integrate different experimental data sets and computational predictions. Other machine learning methods such as deep learning may provide more powerful integration. Third, there is still a big gap between *in vitro* drug activity and clinical phenotype. More data types and modeling techniques, such as quantitative systems pharmacology and pharmacokinetics modeling as well as data mining of electronic health records should be incorporated into the pipeline. With the knowledge of genome-wide target profiles and their associations with diseases, significant time and cost could be saved in the lead optimization, pharmacokinetics, pre-clinical, and other downstream studies using pro-target strategy for drug discovery.

## Materials and methods

### Materials

Solid levosimendan (formula: C_14_H_12_N_6_O, molecular weight: 280.28 g/mol) was purchased from MedChemExpress (New Jersey, USA). The purity of the compound is larger than 98.0% determined by LCMS.

### Kinase binding assay

To validate our computational predictions, we employed a competition binding assay to detect the binding of levosimendan to 425 human kinases as well as its dose-response curve to RIOK1. The proprietary KinomeScan assay was performed by DiscoverX (Fremont, California, USA). The tests were performed at 10 μM and 100 μM concentrations of levosimendan, respectively. Assay results were reported as *%control*, calculated as follows:

$$\%control=\frac{\left(TestCompoundSignal-PositiveControlSignal\right)}{\left(NegativeControlSignal-PositiveControlSignal\right)}\times 100$$

A lower *%control* score indicates a stronger interaction. The KinomeScan experiment and data analysis were performed by DiscoverX.

Binding constant (K_d_) was calculated with a standard dose-response curve using the Hill equation:

$$response=background+\frac{signal-background}{1+(K_{d}{}^{-1}/dose^{-1})}$$

Curves were fitted using a non-linear least square fit with the Levenberg-Marquardt algorithm.

### Cancer cell line assay

OncoPanel cancer cell proliferation assay was performed by Eurofins Panlabs, Inc. (Missouri, USA). Cancer cells were grown in RPMI 1640, 10% FBS, 2 mM L-alanyl-L-glutamine, 1 mM Na pyruvate, or a special medium. Cells were seeded into 384-well plates and incubated in a humidified atmosphere of 5% CO_2_ at 37°C. Compounds were added the day following cell seeding. At the same time, a time zero untreated cell plate was generated. After a 3-day incubation period, cells were fixed and stained to allow fluorescence imaging of nuclei.

Levosimendan was serially diluted in half-log steps from 100 μM and assayed over 10 concentrations with a maximum assay concentration of 0.1% DMSO. Automated fluorescence microscopy was carried out using a Molecular Devices ImageXpress Micro XL high-content imager, and images were collected with a 4X objective. 16-bit TIFF images were acquired and analyzed with MetaXpress 5.1.0.41 software.

Cellular response parameters were calculated using nonlinear regression to a sigmoidal single-site dose response model:

y=A+B−A1+(Cx)D

where *y* is the relative cell count measured following treatment with levosimendan at a concentration *x*. *A* and *B* are the lower and upper limits of the response, *C* is the concentration at the response midpoint (EC_50_), and *D* is the Hill Slope [52].

Cell count EC_50_ is the test concentration at the curve inflection point (parameter *C*), or half the effective response. IC_50_ is the test compound concentration at 50% of the maximum possible response. GI_50_ is the concentration needed to reduce the observed growth by half (midway between the relative cell count at the curve maximum and at the time of compound addition). Activity area is an estimate of the integrated area above the response curve [45]. Activity area values range from 0–10, where a value of zero indicates no inhibition of proliferation at all concentrations, and a value of 10 indicates complete inhibition of proliferation at all concentrations.

### Computational methods

#### Ligand binding site similarity search across human structural proteome

The computational procedure has been reported previously [21–27]. Briefly, we used PDE3B (PDB ID 1SO2) that was the reported molecular target of levosimendan as the template for the binding site analysis. The SMAP software [29–31] was applied to characterize ligand-binding potential from the geometric, physiochemical, and evolutionary characteristics of its binding pocket, and to predict the binding site similarity between the template and 10,472 non-redundant human protein structures. The *p*-value of ligand binding site similarity was normalized by structural classes (e.g. all-alpha, all-beta, and mixed alpha-beta). The structures whose ligand binding sites were predicted to be similar to that of PDE3B with the *p*-value < 0.002 were selected as the initial candidate off-targets of PDE3B inhibitors. Autodock Vina was used to predict the binding energy between selected off-targets and levosimendan, milrinone, anagrelide, amrinone, and enoximone. Drug-target interactions (matrix *Q*) that had docking scores less than -7.5 were remained to be incorporated into the genome-wide chemical protein interactions network in the next step.

#### Genome-wide drug-target prediction

Genome-wide drug-target interactions were predicted using 3D-REMAP. 3D-REMAP takes four networks as input: chemical-protein association (matrix *R*), off-target (matrix *Q*), chemical-chemical similarity, protein-protein similarity networks, The chemical-protein associations were obtained by integrating three resources: 1) publicly available databases, ChEMBL [53] (v23.1) and DrugBank [54] (v5.5.10), 2) four data sets from recent publications about kinome assays [55–58], and 3) protein structure-based off-target prediction from previous step. From ChEMBL, inhibition assays having *IC*_*50*_ ≤ 10 *μM* was regarded as active associations. Those with suboptimal confidence scores (i.e. confidence < 9) were excluded. From DrugBank, drug-target, drug-enzyme, drug-carrier, and drug-transporter associations were collected. The data sets from kinome assays are available in different types of activity measurement. Christmann-Franck *et al*. collected chemical-kinase assays from multiple past publications and presented the activity standardization protocol, which assumed an activity with *K*_*i*_ ≤ 5 *μM* is active [55]. If the original publication presented percent inhibition (or percent remaining activity) at a given compound concentration, *K*_*i*_ was calculated as follows:

$$K_{i}=\frac{concentration\times (100-\%inhibition)}{\%inhibition}$$

If the original publication presented value, *K*_*i*_ was obtained by *K*_*i*_ = 10^-*pKi*^. For this study, we followed the above standardization protocol to integrate kinome assay data with the public databases. We considered chemical-kinase association active if K_i_ ≤ 5 *μM* or *pK*_*i*_≥ 5. To map chemicals from multiple sources, we used OpenBabel to convert all chemical molecules to InChIKey, a 27-character molecular representation developed to help searching chemical molecules. Protein targets were mapped by their UniProt accession. Low confidence targets from reference [57] were excluded. Off-target network was obtained using the procedure described in the previous section of Method. Chemical-chemical and protein-protein similarity scores were calculated similarly in the reference [33]. MadFast software developed by ChemAxon (Budapest, Hungary. https://chemaxon.com/) was used to calculate chemical-chemical similarity matrix, and BLAST was used to calculate protein-protein similarity matrix. The integrated chemical-protein association network contains 650,581 positively associated chemical-protein pairs for 1,656,274 unique chemicals and 9,685 unique target proteins. The chemical-chemical and protein-protein similarity matrices contain 122,421,717 and 31,266 nonzero similarity scores, respectively.

3D-REMAP assumes that chemical and protein space can be represented by two low rank matrices, *U*_*n×r*_ and *V*_*m×r*_, respectively. Given an observed chemical-protein interaction matrix *R*, predicted off-target matrix *Q*, chemical-chemical similarity matrix *C*, and protein-protein similarity matrix *T*, the *U* and *V* are obtained by iteratively minimizing the objective function,

$$\underset{U,V\geq 0}{\text{min}}\sum_{\left(i,j\right)}w{\left(R_{\left(i,j\right)}+Q_{\left(i,j\right)}-U_{\left(i,:\right)}\cdot V_{\left(j,:\right)}^{T}\right)}^{2}+\alpha ({\left\|U\right\|}^{2}+{\left\|V\right\|}^{2})+\beta tr(U^{T}(D_{C}-C)U)+\gamma tr(V^{T}(D_{T}-T)V)$$ (1)

Here, *w* is the confidence weight on the observed and predicted off-target associations which indicate the reliability of the assigned probability of true association; *α* is the regularization parameter to prevent overfitting; *β* is the importance parameter for chemical-chemical similarity, *γ* is the importance parameter for protein-protein similarity, and *tr*(A) is the trace of matrix A. The predicted score for the *i*^*th*^ chemical to bind the *j*^*th*^ protein can be calculated by $P_{\left(i,j\right)}\;=\;U_{UP(i,:)}{∙V}_{UP(j,:)}^{T}$, where *U*_*UP*_ and *V*_*UP*_ are the low-rank matrices *U* and *V* after completion of the updates. Different from original winOCCF [33], *Q* in 3D-REMAP is the predicted off-target network instead of a fixed imputation value. More details on the optimization algorithm of Eq (1) are published elsewhere [33].

#### Gene expression and biological pathway analysis

Cell lines are classified as “sensitive” (activity area ≥ 1.95), “intermediate” (1.3 < activity area < 1.95), or “resistant” (activity area ≤ 1.3). Student’s t-test is used to compare log_2_-transformed mRNA probe levels between sensitive and resistant groups, and a *p*-value is calculated for each probe. The fold change in mRNA expression is calculated as:

$$Fold\;Change=\{\begin{array}{c} \;\;E_{S}/E_{R},\;\;\;if\;E_{S}>E_{R}\\ \;-E_{R}/E_{S},\;\;\;if\;E_{S}<E_{R} \end{array}$$

where *E*_*S*_ and *E*_*R*_ are the mean log_2_ mRNA probe levels for a given gene in cell lines found in the sensitive and resistant groups, respectively.

A false discovery rate (FDR)-adjusted *p*-value (*q*-value) is computed with a null hypothesis of no difference between “sensitive” and “resistant” groups. The *q*-value is calculated according to the following formula:

$$q=(\frac{p}{rank})\times N$$

where *rank* is the rank of the *p*-value and *N* is the number of conducted tests. Gene set over-representative analysis was carried out using DAVID [59].

#### Predictive modeling of levosimendan sensitivity of cancer cell lines and TCGA samples

The gene expression profiles of cell lines in CCLE [45] and GDSC [46] are used as features to build the pharmacogenomics model of levosimendan. Because of the inconsistency in the genomics data between these two data sets, two separate models are developed. 640 genes are identified to be responsible for the drug sensitivity (475 genes) and resistance (165 genes) based on the gene expression profile analysis as described in the previous section. They are used as initial gene set for the machine learning model. 97 and 85 genes are further selected via Kernel Conditional Covariance Minimization [44] for CCLE and GDSC data set, respectively. The gene expression profile of these genes are used to train and test the final models.

Using the gene expression profile of the selected genes as features and the activity area of cancer cell line sensitivity of levosimendan as the target variable, regression models were trained using ElasticNet, Random Forest, Support Vector Regression (SVR), and Gradient Boosting Regression as implemented in Scikit-learn. The features were standardized according to the machine learning algorithms applied. The optimal parameters were determined by grid search, and the performances were evaluated using nested leave-one-out cross-validation. ElasticNet and SVR were chosen as the best performed algorithms for CCLE and GDSC, respectively. After the models were trained, the response of remaining CCLE and GDSC cell lines that were not in the training data to levosimendan were predicted using corresponding models. The response of TCGA samples to levosimendan were predicted using CCLE trained model because the gene expression was measured by RNA-seq in both data sets.

#### Binding pose analysis

For experimentally validated kinase targets of levosimendan RIOK1, the binding pose of levosimendan is predicted using protein-ligand docking software AutodockFR [37] and visualized using DS Visualizer. Solved ADP-bound complex structure (PDB Id: 4OTP) is used for the docking experiment. Co-crystallized water molecules are remained in the docking.

#### Visualization

The kinome tree in Fig 2 is generated from KinMap [60]. The binding poses in Fig 3 is generated from DS Visualizer (Dassault Systèmes BIOVIA, San Diego).

## Supporting information

S1 Table KinomeScan results of levosimendan. (CSV)

S2 Table Protein-ligand docking results of levosimendan across human kinome. (CSV)

S3 Table Off-targets of levosimendan which were determined by ligand similarity search. (CSV)

S4 Table OncoPanel results of levosimendan against 200 cancer cell lines. (XLSX)

S5 Table Differential gene expression profile analysis for the sensitivity and resistance of cancer cell lines responding to the treatment of levosimendan. (XLSX)

S6 Table Predicted active area of cell lines under the treatment of levosimendan. (XLSX)

S7 Table Predicted active area of patients in TCGA under the treatment of levosimendan. (XLSX)
